# A Systematic Review of Economic Evaluations of Active Tuberculosis Treatments

**DOI:** 10.3389/fphar.2021.736986

**Published:** 2021-12-13

**Authors:** Joo-Young Byun, Hye-Lin Kim, Eui-Kyung Lee, Sun-Hong Kwon

**Affiliations:** ^1^ School of Pharmacy, Sungkyunkwan University, Suwon, South Korea; ^2^ College of Pharmacy, Sahmyook University, Seoul, South Korea

**Keywords:** tuberculosis, economic evaluation, cost-effectiveness analysis, cost-utility analysis, systematic review

## Abstract

**Background:** The disease burden of active tuberculosis (TB) is considerable, but systematic reviews of economic evaluations of active TB treatments are scarce.

**Methods:** PubMed, EMBASE, and the Cochrane Library databases were used to search for articles on cost-effectiveness analysis or cost-utility analysis that economically evaluated active TB treatments, which were then systematically reviewed by two independent reviewers. We extracted vital components of the included studies, such as country, population, intervention/comparator, primary outcome, values of outcomes, thresholds, model type, time horizon, and health states included in the model.

**Results:** Seventeen studies were included in this systematic review. Thirteen dealt with interventions of medications, and the remaining four compared care strategies. The Markov model was the most commonly used tool to compare medications, whereas studies on care plans mainly used decision trees. The most commonly used primary outcome was disability-adjusted life years, followed by quality-adjusted life years. For treatment-naïve TB, the 4-month regimen was more cost-effective than the 6-month regimen mainly in low- and middle-income countries. For multidrug-resistant TB, a bedaquiline-based regimen was cost-effective. For multidrug-resistant TB, decentralized care that employed the use of home or mobile devices was more cost-effective than hospital-based centralized care in low- and middle-income countries.

**Conclusion:** New treatment strategies to improve therapeutic outcomes by enhancing treatment adherence, such as regimens with shorter durations (2 or 4 months) and decentralized care, or new anti-TB agents (e.g., bedaquiline) have been suggested as cost-effective interventions for active TB. This review provides information on the economic evaluation of active TB from good-quality studies, thus aiding the future economic evaluation of active TB.

## 1 Introduction

Tuberculosis (TB) is an infectious disease that has long been among the top 10 major causes of death worldwide. *Mycobacterium tuberculosis*, the causative bacteria of TB, is the leading cause of death among infectious agents, with an estimated 10 million diagnoses of TB annually (WHO, 2019). Once *Mycobacterium tuberculosis* is infected in human body, it evolves from latent TB to active TB. At the stage of latent TB, it is asymptomatic and not transmissible since the pathogen is isolated in the granuloma. However, once it is evolved into active TB, patients have symptoms such as fever, cough, or fatigue and it usually becomes diagnosable with sputum smear, culture and molecular tests. Likewise, active and latent TB have different traits, and therefore, diagnostics and treatment strategies differ between active and latent TB (Pai et al., 2016).

There have been many challenges in treating active TB, including low cure rates with the standard regimen due to its long duration, severe toxicity, and poor medication adherence (Ernest et al., 2020). Treatments are difficult for multidrug-resistant tuberculosis (MDR-TB) with more cocktails of medications over 2 years and daily injections that cause severe side effects (O'Neill, 2016). Approximately 18% of TB patients who were previously treated with a standard regimen have MDR-TB or rifampicin-resistant TB (WHO, 2019). MDR-TB and extremely drug-resistant tuberculosis (XDR-TB) cause an estimated 200,000 deaths annually (O'Neill, 2016). The disease burden of active TB was also revealed in a previous retrospective cohort study showing higher mortality and healthcare resource utilization in an active TB cohort than a non-active TB cohort (Wada et al., 2020). Considering the growing requirement for new drugs to combat drug resistance (Dover et al., 2008) and new strategies to improve low treatment adherence (Munro et al., 2007; Bea et al., 2021), several new medications/regimens or new care strategies for active TB treatments have been developed. From a societal viewpoint, it is necessary to find cost-effective treatments, making it necessary to conduct economic evaluations.

Previous studies have focused on economic evaluations for latent TB (Campbell et al., 2015; Zammarchi et al., 2015; Auguste et al., 2016; Marks et al., 2017; Greenaway et al., 2018a) or active and latent TB patients simultaneously (Chavan et al., 2011; Verdier et al., 2011; Silva et al., 2018). One systematic review focused on active TB patients but analyzed only the screening of active TB (Greenaway et al., 2018b). In addition, systematic reviews on economic evaluations of diagnosis (Sagili et al., 2018; Hao et al., 2020) and vaccination (Machlaurin et al., 2019) of TB have been published. However, systematic reviews focusing on the economic evaluation of treatments for patients with active TB are scarce. Reviewing methodologies and analyzing published results of recent economic evaluations on active TB treatments would help better understand the current flow of developing interventions for active TB and their cost-effectiveness status. Therefore, this study aimed to conduct a comprehensive systematic review of economic evaluations of medications/regimens or care strategies for active TB patients published over the past 10 years.

## 2 Materials and Methods

This systematic review was conducted following the Preferred Reporting Items for Systematic Reviews and Meta-Analyses guidelines (Page et al., 2021).

### 2.1 Eligibility Criteria

We included studies that conducted an economic evaluation of anti-TB medications or care strategies for active TB. To include active TB patients, studies that included at least one arm of patients treated with anti-TB medication were included. Studies in which subjects were only animals or patients with non-active TB (e.g., latent TB) were excluded. If an article included active and latent TB patients simultaneously with separately presented results of active and latent TB, the article was selected. Studies evaluating the screening and diagnosis of and vaccination for TB were excluded. Articles published before 2010 or abstracts without full text were excluded.

### 2.2 Search Strategy and Selection of Studies

We searched for studies published between January 1, 2010, and January 29, 2020, that economically evaluated active TB treatment, using the PubMed, EMBASE, and Cochrane Library databases. Considering each database system, we combined terms related to the participants of interests and study types using Medical Subject Headings and title and abstract (tiab) as search fields. The search terms were as follows: the Medical Subject Headings term of “tuberculosis,” “Cost-Benefit Analysis,” and the text string of “cost,” “effective*,” “utility,” and “model.” The complete search strategy used for each database is included in Supplementary Table S1. The process of selecting or excluding studies was carried out using Endnote software (Clarivate, London, United Kingdom) as a citation manager. After excluding duplicated articles, two reviewers (JY, MJ) screened the titles and abstracts of all the remaining articles according to the eligibility criteria. Two other reviewers (SH, HL) reviewed the full texts of the retrieved articles to determine whether they met the eligibility criteria. In cases of disagreement between reviewers, a consensus was achieved through discussion.

### 2.3 Data Extraction and Analysis

Using an Excel sheet, two reviewers (JY, MJ) independently extracted information from selected articles and discussed discrepancies during cross-checking. Data on the following characteristics of the included studies were extracted: target population, type of intervention, country, primary outcome, model type, time horizon, and health states included in the model. In addition, we extracted data on the key components of each economic evaluation using a predefined extraction format. The extracted components were intervention/comparator, outcome type, outcome values, cost-effectiveness thresholds, cost-effectiveness status of the outcomes, and funding information. We organized the status of cost-effectiveness of each outcome using stated thresholds or the author’s statements on cost-effectiveness. Two other reviewers (SH and HL) screened all the extracted data to confirm it was complete and accurate. Each cost-related outcome, based on various currencies depending on the country of the study, was converted into US dollars using the exchange rate of July 2020.

### 2.4 Quality Assessment

To assess the methodological quality of all the selected studies, we used the Quality of Health Economic Studies (QHES) instrument, which has been validated for appraising the methodological quality of economic evaluations (Joshua et al., 2003). The QHES instrument includes 16 criteria, and each criterion has a weighted point value, ranging from 1 to 9, allowing users to estimate total values up to 100. Two reviewers (JY, MJ) evaluated all the included studies using the QHES instrument and resolved any disagreements through discussion and consensus. We allowed an intermediate interpretation; we evaluated performance on a criterion as “partial” when part of the criterion was fulfilled but another was unfulfilled.

## 3 Results

### 3.1 Included Studies and Settings

**FIGURE 1:**
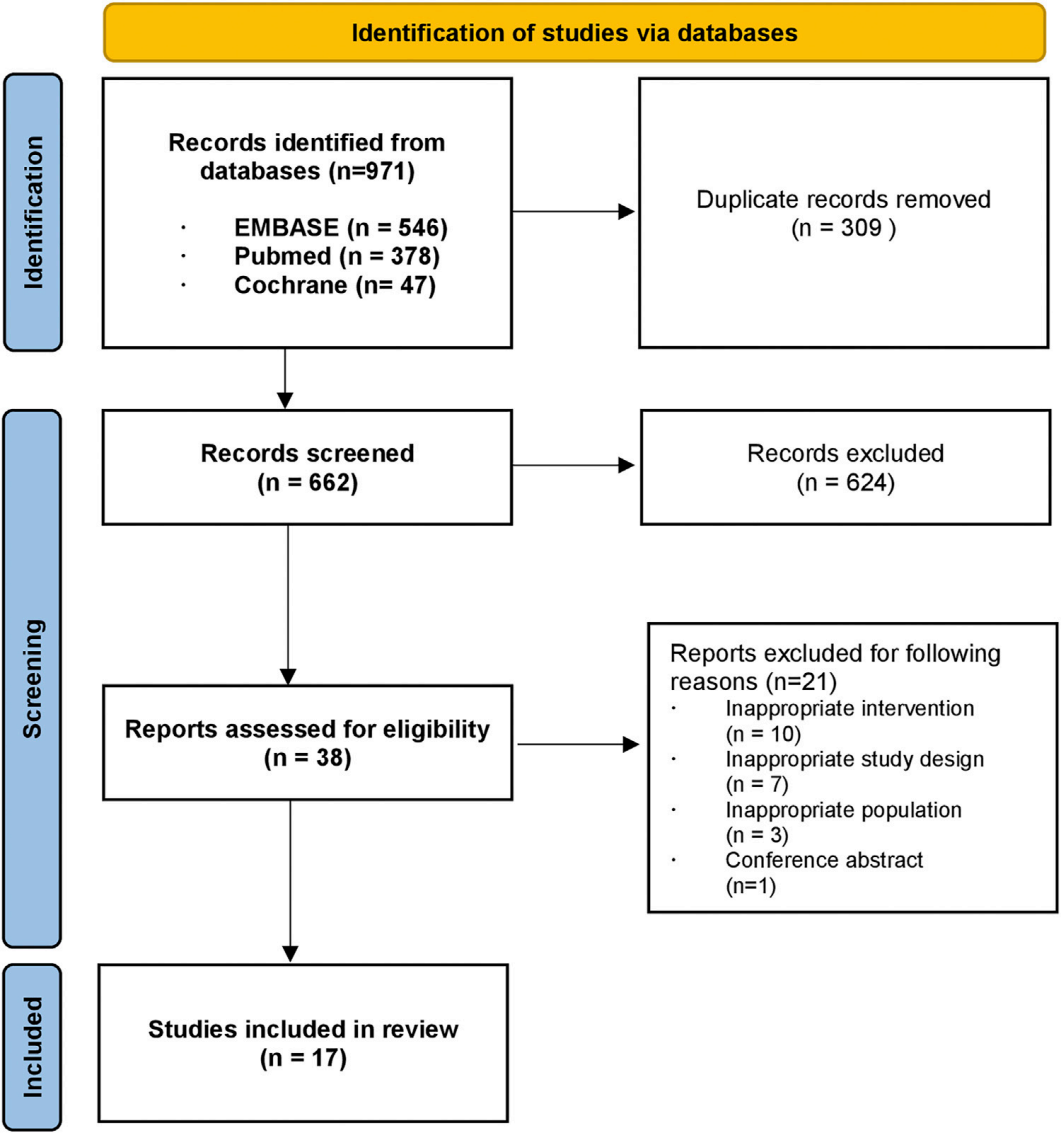
Flow chart of the study selection process.

A total of 971 citations were identified through a database search. After excluding 309 duplicates, we screened 662 articles by title and abstract, leaving 38 articles for full-text screening. Through full-text screening, 17 articles were eligible for inclusion. A flow chart of the study selection process is presented in Figure 1. The basic characteristics of the 17 included studies are summarized in Table 1. Two studies were carried out in more than one country (Gomez et al., 2016; Lu et al., 2017); however, most (15 out of 17) were carried out in one country. Ten studies included results from low- and middle-income countries (LMICs) according to World Bank income classification and 7 of them (Manabe et al., 2012; Knight et al., 2015; Gomez et al., 2016; Lu et al., 2017; Schnippel et al., 2018a; Schnippel et al., 2018b; Loveday et al., 2018) were conducted in sub-Saharan Africa. Lu et al. (2017) conducted a cost-effectiveness analysis of seven countries specified as having a high burden of TB in the World Health Organization Global Tuberculosis Report 2014 (WHO, 2014), which include a high-income country (HIC) (Estonia) and LMICs simultaneously, to examine the health outcomes of novel treatment. Gomez et al. (2016) estimated the cost and cost-effectiveness of TB treatments in four different countries, two of which were middle-income countries, and the other two were low-income countries.

**TABLE 1:** Characteristics of the included studies (*N* = 17).

Study	Population	Type of intervention	Country	Primary outcome	Model type	Time horizon (Years)
MDR-TB						
Codecasa et al. (2017)	MDR-TB; XDR-TB	Drug	Italy (HIC)	LYG	Markov model	10
Lu et al. (2017)	MDR-TB; XDR-TB	Drug	Selected high-burden countries: Estonia (HIC), Russia, South Africa, Peru, China, the Philippines, and India (LMICs)	DALY; successful outcome; acquired resistance	Markov model	10
Park et al. (2016)	MDR-TB; XDR-TB	Drug	South Korea (HIC)	QALY; LYG	Markov model	20
	HIV−					
Schnippel et al. (2018a):1	MDR-TB; XDR-TB; RR-TB	Drug	South Africa (LMIC)	DALY	Markov model	10
	HIV+/−					
Schnippel et al. (2018b):2	MDR-TB; RR-TB	Drug	South Africa (LMIC)	DALY	Markov model	10
	HIV+/−					
Fan et al. (2019)	MDR-TB	Drug	China (Hong Kong) (HIC)	QALY	Decision tree + Markov model	10 (2 + 8)
John et al. (2018)	MDR-TB	Care	India (LMIC)	QALY	Decision tree	2
	HIV−					
Loveday et al. (2018)	MDR-TB	Care	South Africa (LMIC)	Treatment success rate	Observational study	5
	HIV+/−					
Wirth et al. (2017)	MDR-TB	Drug	Germany (HIC)	QALY	Markov model	10
Wolfson et al. (2015)	MDR-TB	Drug	The United Kingdom (HIC)	QALY; DALY	Markov model	10
Drug-susceptible/Treatment-naïve TB						
Gomez et al. (2016)	Treatment naïve TB	Drug	South Africa, Brazil, Bangladesh, Tanzania (LMICs)	DALY	Individual-based decision-analytic model	Lifetime
	HIV+/−					
Hunchangsith et al. (2012)	Treatment naïve TB	Care	Thailand (LMIC)	DALY	Decision tree	Lifetime
	HIV−					
Knight et al. (2015)	Treatment naïve TB	Drug	South Africa (LMIC)	DALY; TB case; Death	Transmission model	20
	HIV+/−					
Law et al. (2014)	Treatment naïve TB	Drug	Canada (HIC)	DALY; MDR case; Death	Markov model	10
	HIV+/−^a^					
Manabe et al. (2012)	Treatment naïve TB	Drug	Uganda (LMIC)	Death	Decision tree	2.5
	HIV+/−					
Owens et al. (2013)	Treatment naïve TB	Drug	The United States (HIC)	DALY	Decision tree	Lifetime
MDR and drug-susceptible/treatment-naïve TB						
Nsengiyumva et al. (2018)	- MDR TB	Care	Brazil (LMIC)	DALY	Decision tree	4
	- drug-susceptible					

MDR-TB, multidrug-resistant tuberculosis; XDR-TB, extremely drug-resistant tuberculosis; RR-TB, rifampicin-resistant tuberculosis; HIV, human immunodeficiency virus; LYG, life-years gained; DALY, disability-adjusted life years; QALY, quality-adjusted life years; HIC, high-income country; LMIC, low- and middle-income country.

a Due to scarcity of parameters specific to TB patients with concomitant HIV, the study assumed similar treatment outcomes between HIV positive and negative patients.

Five studies conducted cost-effectiveness analyses using various scenarios or settings, including different treatment costs, and durations (Owens et al., 2013), populations with different percentages of drug resistance using several types of outcomes (Law et al., 2014), different countries with different levels of adherence to guidelines (Gomez et al., 2016) or outcome types (Lu et al., 2017), and different TB cohorts and perspectives (Nsengiyumva et al., 2018).

### 3.2 Population and Interventions

**FIGURE 2:**
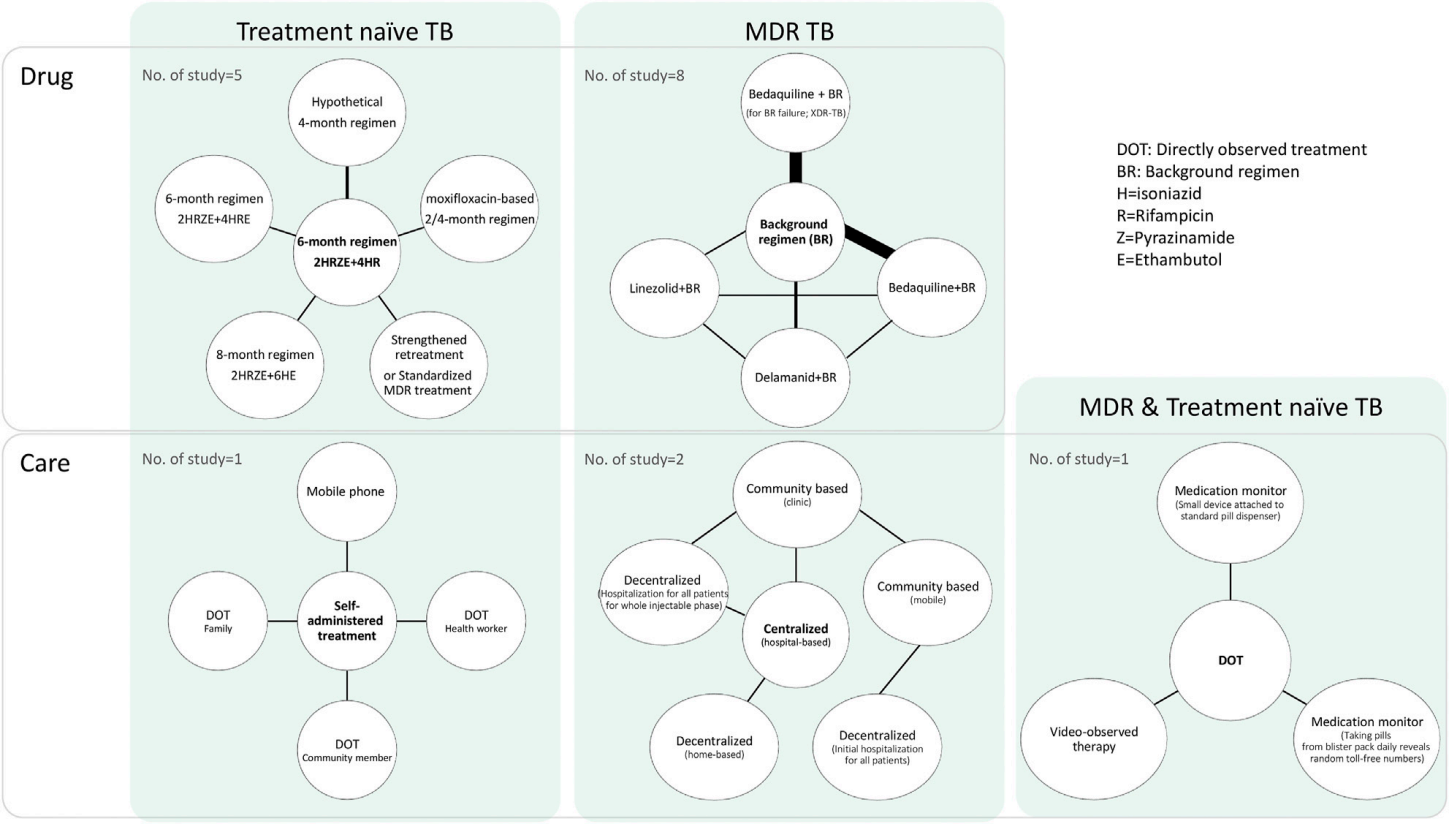
Network diagram of the interventions and study populations.

Figure 2 presents a network diagram of the interventions and study populations. In terms of the study population, 7 studies (Hunchangsith et al., 2012; Manabe et al., 2012; Owens et al., 2013; Law et al., 2014; Knight et al., 2015; Gomez et al., 2016; Nsengiyumva et al., 2018) were conducted with drug-susceptible or treatment-naïve TB (hereafter treatment-naïve TB) patients who initiated TB treatment for the first time. Eleven studies were conducted with MDR-TB patients (Wolfson et al., 2015; Park et al., 2016; Codecasa et al., 2017; Lu et al., 2017; Wirth et al., 2017; Schnippel et al., 2018a; Schnippel et al., 2018b; John et al., 2018; Loveday et al., 2018; Nsengiyumva et al., 2018; Fan et al., 2019). Nsengiyumva et al. (2018) conducted a study with treatment-naïve and MDR-TB patients using two distinct models and included latent TB patients. Six out of 8 studies which were conducted in HICs were dealing with MDR-TB patients (Wolfson et al., 2015; Park et al., 2016; Codecasa et al., 2017; Lu et al., 2017; Wirth et al., 2017; Fan et al., 2019), whereas studies from LMICs were relatively balanced with MDR-TB (6 studies) (Lu et al., 2017; Schnippel et al., 2018a; Schnippel et al., 2018b; John et al., 2018; Loveday et al., 2018; Nsengiyumva et al., 2018) and treatment-naïve TB (5 studies) (Hunchangsith et al., 2012; Manabe et al., 2012; Knight et al., 2015; Gomez et al., 2016; Nsengiyumva et al., 2018).

In terms of intervention types, most of the selected articles (13 out of 17) examined interventions of medications, comparing the standard 6-month regimen with longer (8-month) (Manabe et al., 2012) or shorter regimens (2 or 4 months) (Owens et al., 2013; Knight et al., 2015; Gomez et al., 2016) in treatment-naïve TB patients, or compared background regimens with new regimens that contain new drugs, such as bedaquiline, in MDR-TB patients (Wolfson et al., 2015; Park et al., 2016; Codecasa et al., 2017; Lu et al., 2017; Wirth et al., 2017; Schnippel et al., 2018a; Schnippel et al., 2018b; Fan et al., 2019). One study (Law et al., 2014) compared standardized TB treatments with varying drug resistance in treatment-naïve patients with TB.

The other four articles examined “care” types, which were generally related to new methods of improving patient medication adherence, including comparing self-administered treatment or directly observed treatment (DOT) with other types of care in treatment naïve TB patients (Hunchangsith et al., 2012; Nsengiyumva et al., 2018) or comparing hospital-based centralized care with decentralized care in MDR-TB patients (John et al., 2018; Loveday et al., 2018). In a simulation study, Nsengiyumva et al. (2018) compared DOT with various monitoring strategies in treatment-naïve and MDR-TB patients.

### 3.3 Economic Evaluation Model

Table 1 presents the components of the economic evaluation model. Nine out of 17 studies used a Markov model (Law et al., 2014; Wolfson et al., 2015; Park et al., 2016; Codecasa et al., 2017; Lu et al., 2017; Wirth et al., 2017; Schnippel et al., 2018a; Schnippel et al., 2018b; Nsengiyumva et al., 2018; Fan et al., 2019), and 6 studies used a decision tree model (Hunchangsith et al., 2012; Manabe et al., 2012; Owens et al., 2013; John et al., 2018; Nsengiyumva et al., 2018; Fan et al., 2019). Fan et al. (2019) conducted an economic evaluation using a decision tree combined with a Markov model. Distinctively, Knight et al. (2015) used a transmission model, and Gomez et al. (2016) used an individual-based decision-analytic model. Loveday et al. (2018) conducted a study without stating an economic model. Three out of four studies that compared care types used a decision tree model (Hunchangsith et al., 2012; John et al., 2018; Nsengiyumva et al., 2018), while most studies (9 out of 13) that compared drugs or regimens used a Markov model (Law et al., 2014; Wolfson et al., 2015; Codecasa et al., 2017; Lu et al., 2017; Wirth et al., 2017; Schnippel et al., 2018a; Schnippel et al., 2018b; Fan et al., 2019).

The most frequently used primary outcome was disability-adjusted life years (DALYs), used in 10 studies (Hunchangsith et al., 2012; Owens et al., 2013; Law et al., 2014; Knight et al., 2015; Wolfson et al., 2015; Gomez et al., 2016; Lu et al., 2017; Schnippel et al., 2018a; Schnippel et al., 2018b; Nsengiyumva et al., 2018). This was followed by quality-adjusted life years (QALYs), used in five studies (Wolfson et al., 2015; Park et al., 2016; Wirth et al., 2017; John et al., 2018; Fan et al., 2019). Wolfson et al. (2015) used DALYs and QALYs. Other outcomes included death (3 studies) (Manabe et al., 2012; Law et al., 2014; Knight et al., 2015), life-years gained (LYGs) (2 studies) (Park et al., 2016; Codecasa et al., 2017) and new TB cases (2 studies) (Law et al., 2014; Knight et al., 2015).

The time horizon varied across studies, from 2 years (John et al., 2018) to a lifetime (Hunchangsith et al., 2012; Owens et al., 2013; Gomez et al., 2016). Studies that used the Markov model tended to have longer time horizons (e.g., 10 or 20 years) compared with studies that used decision tree models (e.g., 2, 4, or 5 years), although two studies that used decision tree models had time horizons of a lifetime (Hunchangsith et al., 2012; Owens et al., 2013).

Among the health states included in the models for each TB population, studies generally included health states related to cure/fail, lost to follow-up (defaulted), treatment completion, and retreatment for MDR-TB and treatment naïve TB. However, health states related to sputum conversion, end-of-life/palliative care, comorbidity of human immunodeficiency virus (HIV), and surgery were found in models for MDR-TB but rarely found in models for treatment-naïve TB patients.

Six out of 7 studies which were conducted in sub-Saharan Africa were reflecting HIV status in their economic evaluation models. The 6 economic evaluation models used treatment outcomes according to HIV status and included cost for HIV treatment. Schnippel et al. (2018a; 2018b) included health states of HIV treatment such as “initiate HIV treatment” and “continue HIV treatment” in their Markov models. Loveday et al. (2018) adjusted for baseline variables including HIV and antiretroviral therapy (ART) status between 5 cohorts using propensity score weighting with a prospective cohort design. Manabe et al. (2012) divided the patients into HIV-positive and HIV-negative arm in decision tree model to reflect high co-infection of HIV and TB in Uganda. Gomez et al. (2016) used ART coverage level of five different countries as a scenario parameter using individual-based decision analytic model. The Characteristics of TB such as duration and smear positive rates differed by HIV status in Knight et al. (2015) which used transmission model.

To establish an economic evaluation model that is generally simulated by a hypothetical cohort, most of the included studies earned treatment outcome for each intervention and comparator mainly from published data of clinical trials, but Loveday et al. (2018) earned the treatment outcome from prospective cohort analysis, by observing the outcome from 5 cohorts which were allocated with 5 care strategies. In most included studies, safety data of the drugs was reflected in the economic evaluation models since data from clinical trials implicit safety data. Particularly, five studies mentioned how they reflected safety data on their economic evaluation models. Law et al. (2014) included blind health state in their Markov model to reflect ocular toxicity of ethambutol. Wolfson et al. (2015) modeled differences in safety between two strategies in their Markov model using data from randomized trial. Schnippel et al. (2018a) analyzed cost needed for screening adverse reaction. Schnippel et al. (2018b) compared cost-effectiveness with or without considering adverse reactions. Wirth et al. (2017) included cost for adverse events with detailed incidence and cost for each adverse reaction of medications.

### 3.4 Cost-Effectiveness

Table 2 shows the results of the comparison of the regimens for drug-susceptible/treatment-naïve TB patients. In three studies which were conducted in LMICs, shortened regimens were more cost-effective than regimens with longer duration. The 6-month standard regimen was more cost-effective than an 8-month regimen (Manabe et al., 2012). A hypothetical 4-month regimen was more cost-effective than 6-month standard regimen in most (7 out of 9) comparisons (Knight et al., 2015; Gomez et al., 2016), but in Bangladesh, it was not more cost-effective than the 6-month standard regimen (Gomez et al., 2016). The cost-effectiveness of other new regimens was assessed in HICs. A strengthened retreatment regimen (regimen with second-line drugs for patients who failed or relapsed after cure or default) was more cost-effective than 6-month standard regimen in all comparisons (Law et al., 2014). A moxifloxacin-based 4-month regimen was more cost-effective than a 6-month standard regimen, whereas a moxifloxacin-based 2-month regimen was less costly and more effective in settings with high treatment costs, but it was not possible to assess cost-effectiveness in settings with moderate/low treatment costs because the exact amount of threshold was not suggested (Owens et al., 2013). Ethambutol-added regimen (2 months of isoniazid, rifampicin, pyrazinamide, and ethambutol, followed by 4 months of isoniazid, rifampicin, and ethambutol) was more cost-effective than the 6-month standard regimen when MDR case and TB death were used as outcomes but less effective when DALY was used as the outcome. Standardized MDR treatment (regimen received by patients who failed initial treatment for 24 months) was cost-effective when the outcome was DALY, but cost-effectiveness could not be assessed when MDR case and TB death were used as outcomes due to lack of threshold (Law et al., 2014).

**TABLE 2:** Cost-effectiveness of interventions in studies comparing regimens/drugs of drug susceptible/treatment-naïve TB patients

Intervention	Comparator	No. of comparisons	% Cost-effective	% Not cost-effective	% Not available
6-month regimen (2HRZE + 4HR)	8-month regimen (2HRZE + 6HE)	**2 comparisons in Manabe (2012) [Uganda-LMIC]** The regimen with 4HR for continuation treatment was a dominant strategy compared to the regimen with 6HE for continuation treatment	100% (2/2)	–	–
Hypothetical 4-month regimen	6-month regimen (2HRZE + 4HR)	**8 comparisons in Gomez (2016) [South Africa, Brazil, Bangladesh, Tanzania-LMICs]** Cost-effectiveness was estimated according to country and guideline adherence. The 4-month regimen was not cost-effective in Bangladesh for both guideline-adhered scenario and the current scenario because the ICER exceeded the threshold **1 comparison in Knight (2015) [South Africa-LMIC]** The article presented cost-effective cost of 4-month regimen	78% (7/9)	22% (2/9)	–
Strengthened retreatment	6-month regimen (2HRZE + 4HR)	**12 comparisons in Law (2014) [Canada-HIC]** Two regimens were compared in populations with different percentages of isoniazid-resistant and MDR-TB patients using three kinds of outcomes (DALYs, MDR cases, TB death). Strengthened retreatment was applied to patients who failed or relapsed after cure or default with second-line drugs (2 months of daily levofloxacin, rifampicin, pyrazinamide, ethambutol, and streptomycin, followed by 1 month of daily levofloxacin, rifampicin, pyrazinamide, and ethambutol, then by 6 months of thrice-weekly levofloxacin, rifampicin, and ethambutol). Strengthened retreatment dominated standard strategy in all settings	100% (12/12)	–	–
Moxifloxacin-based 2/4-month regimen	6-month regimen (2HRZE + 4HR)	**6 comparisons in Owen (2013) [The United States-HIC]** Cost-effectiveness was estimated according to regimen duration (2 or 4 months) and treatment costs (high/moderate/low). The 4-month regimen was stated as cost-effective in all settings. The 2-month regimen was less costly and more effective in a high treatment cost setting. However, it was not available to assess cost-effectiveness in moderate/low treatment cost settings because the exact amount of threshold was not suggested	67% (4/6)	–	33% (2/6)
6-month regimen (2HRZE + 4HRE)	6-month regimen (2HRZE + 4HR)	**12 comparisons in Law (2014) [Canada-HIC]** Two regimens were compared in populations with different percentages of isoniazid-resistant and MDR-TB patients using three kinds of outcomes (DALY, MDR case, TB death). An Ethambutol added regimen was dominant when MDR case and TB death were used as outcomes (8 comparisons) but less effective when the outcome was DALY (4 comparisons) compared to the standard regimen	67% (8/12)	33% (4/12)	–
Standardized MDR treatment	6-month regimen (2HRZE + 4HR)	**12 comparisons in Law (2014) [Canada-HIC]** Two regimens were compared in populations with different percentages of isoniazid-resistant and MDR-TB patients using three kinds of outcomes (DALY, MDR case, TB death). Patients who failed initial treatment received standardized MDR treatment for 24 months. The standardized regimen was stated as cost-effective when the outcome was DALY (4 comparisons). However, it was not available to assess cost-effectiveness when MDR case and TB death were used as outcomes due to lack of threshold (8 comparisons)	33% (4/12)	–	67% (8/12)

TB, tuberculosis; DALY, disability-adjusted life year; MDR, multi-drug resistant; H, isoniazid; R, rifampicin; Z, pyrazinamide; E, ethambutol; LMIC, low- and middle-income country; HIC, high-income country.

The cost-effectiveness of regimens for MDR-TB patients is presented in Table 3. Three studies included comparison between bedaquiline-based regimen and background regimen in LMICs. For MDR-TB patients who failed the background regimen or for XDR-TB patients, bedaquiline-based regimen was cost-effective (Lu et al., 2017; Schnippel et al., 2018a), but for MDR-TB patients, the cost-effectiveness of bedaquiline-based regimens differed according to the toxicity profile. Bedaquiline-based regimen was less costly and more effective than injection-based regimens when adjusted for toxicity profile in terms of adverse drug reactions but costlier and less effective when adverse drug reactions were not considered (Schnippel et al., 2018b). Six studies assessed regimens with new drugs in HICs. For both MDR- and XDR-TB patients, bedaquiline-based regimens were more cost-effective than background regimen in 6 HICs (Wolfson et al., 2015; Park et al., 2016; Codecasa et al., 2017; Lu et al., 2017; Wirth et al., 2017; Fan et al., 2019). In Germany, bedaquiline-based regimen was more cost-effective than delamanid or linezolid-based regimens, whereas delamanid-based regimens were more cost-effective than linezolid-based regimens (Wirth et al., 2017). Bedaquiline-based regimen was more effective and less costly (Lu et al., 2017; Schnippel et al., 2018a) or had an incremental cost-effectiveness ratio (ICER) lower than the threshold (Park et al., 2016; Codecasa et al., 2017; Schnippel et al., 2018a) when compared to the standard regimen. Linezolid or delamanid-based regimens are not cost-effective compared to the background regimen (Wirth et al., 2017; Fan et al., 2019). Lu et al. (2017) included cost-effectiveness results from both HIC and LMICs. As a HIC, Estonia had the largest thresholds and probability of bedaquiline plus background being cost-effective was highest when highest price was applied. Regarding concomitant HIV status, Schnippel et al. (2018a) conducted sensitivity analysis by changing proportion of HIV positive patients, showing higher ICER with higher proportion. Gomez et al. (2016) showed lower ICER with higher survival in ART in South Africa, Bangladesh, and Tanzania, but the opposite result in Brazil.

**TABLE 3:** Cost-effectiveness of interventions in studies comparing regimens for MDR-TB patients.

Intervention	Comparator	No. of comparisons	% Cost-effective	% Not cost-effective	% Not available
Bedaquiline + BR (for BR failure, XDR-TB)	BR	**2 comparisons in Schnippel (2018):1 [South Africa-LMIC]** Two bedaquiline-based regimens were compared than the standard regimen. Regimen 1 (capreomycin replaced with bedaquiline) was less costly and more effective than the standard regimen. ICER between regimen 2 (kanamycin replaced with bedaquiline) and standard regimen was lower than the threshold	100% (2/2)	–	**–**
		**21 comparisons in Lu (2017) [Russia, South Africa, Peru, China, the Philippines, and India-LMICs], [Estonia-HIC]** Cost-effectiveness was estimated according to country and outcome types (DALY, successful outcomes, acquired resistance). Bedaquiline added regimen improved health outcomes and reduced costs **2 comparisons in Codecasa (2017) [Italy-HIC]** ICER had values lower than the threshold in NHS and societal perspectives **2 comparisons in Park (2016) [South Korea-HIC]** Cost-effectiveness was estimated using two types of outcomes (QALY, LYG). The combination of bedaquiline and standard regimen was a cost-effective option for MDR/XDR-TB than the standard regimen	100% (25/25)	**–**	**–**
Bedaquiline + BR	BR	**2 comparisons in Schnippel (2018):2 [South Africa-LMIC]** The bedaquiline-based regimen was less costly and more effective than the injection-based regimen when adjusted for toxicity profile but more costly and less effective when toxicity profile was not considered.	50% (1/2)	50% (1/2)	–
		**1 comparison in Fan (2019) [China (Hong Kong)-HIC]** ICER comparing bedaquiline plus BR and BR was lower than the threshold **1 comparison in Wirth (2017) [Germany-HIC]** The bedaquiline plus background regimen was stated as the most cost-effective strategy**2 comparisons in Wolfson (2015) [The United Kingdom-HIC]** Cost-effectiveness was assessed using two kinds of outcomes (DALY, QALY). In both cases, the bedaquiline plus background regimen was less costly and more effective than background regimen	100% (4/4)	–	–
Bedaquiline + BR	Delamanid + BR	**1 comparison in Wirth (2017) [Germany-HIC]** The incremental cost utility ratio (Cost/QALY) between bedaquiline plus BR and delamanid plus BR was lower than the suggested threshold. Bedaquiline plus BR was the most cost-effective treatment strategy	100% (1/1)	–	–
Bedaquiline + BR	Linezolid + BR	**1 comparison in Wirth (2017) [Germany-HIC]** The incremental cost utility ratio (Cost/QALY) between bedaquiline plus BR and linezolid plus BR was lower than the suggested threshold. Bedaquiline plus BR was the most cost-effective treatment strategy	100% (1/1)	–	–
Delamanid + BR	Linezolid + BR	**1 comparison in Wirth (2017) [Germany-HIC]** The incremental cost utility ratio (Cost/QALY) between delamanid plus BR and linezolid plus BR was lower than the suggested threshold	100% (1/1)	–	–
Linezolid + BR	BR	**1 comparison in Wirth (2017) [Germany-HIC]** Linezolid and delamanid were dominated by combinations of BR alone and BR plus bedaquiline	–	100% (1/1)	–
Delamanid + BR	BR	**1 comparison in Fan (2019) [China (Hong Kong)-HIC]** ICER comparing delamanid plus background regimen and background regimen was higher than the threshold **1 comparison in Wirth (2017) [Germany-HIC]** It was stated that linezolid and delamanid were dominated by combinations of BR alone and BR plus bedaquiline	–	100% (2/2)	–

MDR, multi-drug resistant; XDR, extremely drug-resistant; TB, tuberculosis; BR, background regimen; QALY, quality-adjusted life year; DALY, disability-adjusted life year; ICER, incremental cost-effectiveness ratio; LMIC, low- and middle-income country; HIC, high-income country.

Table 4 presents the results of the comparisons between care types for active TB patients. All the four studies were conducted in different LMICs. For treatment-naïve TB patients, DOT and mobile phone-based care were compared to self-administered treatment in Thailand, and it was not possible to assess their cost-effectiveness because all the values had uncertainty ranges that crossed zero, which indicates statistical insignificance (Hunchangsith et al., 2012). For MDR-TB patients, decentralized care provided from home (John et al., 2018) or the hospital (Loveday et al., 2018) was more cost-effective than hospital-based centralized care in India and South Africa, respectively. In particular, community-based care using mobile devices was more cost-effective than hospital-based decentralized care or community-based care in clinics (Loveday et al., 2018). For treatment-naïve and MDR-TB patients, video-observed therapy or medication monitoring with devices or blister packs were more cost-effective than DOT in Brazil (Nsengiyumva et al., 2018).

**TABLE 4:** Cost-effectiveness of interventions in studies comparing types of care for active TB patients.

Intervention	Comparator	No. of comparisons	% Cost-effective	% Not cost-effective	% Not available
Drug-susceptible/Treatment naïve TB					
DOT by a health worker	Self-administered treatment	**4 Comparisons in Hunchangsith (2012) [Thailand-LMIC]** For all interventions, cost-effectiveness was not available to assess due to wide uncertainty ranges that crossed zero.	–	–	100% (1/1)
DOT by a community member			–	–	100% (1/1)
DOT by a family member			–	–	100% (1/1)
Mobile phone (contact-reminder system)			–	–	100% (1/1)
MDR-TB					
Decentralized care (home-based)	Centralized care (hospital-based)	**1 Comparison in John (2018) [India-LMIC]** The incremental cost and utility ratio between home-based decentralized care and hospital-based centralized care was lower than the threshold.	100% (1/1)	–	–
Decentralized care (hospitalization for all patients for whole injectable phase)	Centralized care (hospital-based)	**4 Comparisons in Loveday (2018) [South Africa-LMIC]** The success rate increased, and the cost decreased in decentralized care (hospitalization for all patients for whole injectable phase) compared with hospital-based centralized care and clinic compared with decentralized care (hospitalization for all patients for whole injectable phase). The mobile model was the most cost-effective in the article.	100% (1/1)	–	–
Community-based (clinic)	Decentralized care (hospitalization for all patients for whole injectable phase)		100% (1/1)	–	–
Community-based (mobile)	Community-based (clinic)		100% (1/1)	–	–
Community-based (mobile)	Decentralized care (initial hospitalization for all patients)		100% (1/1)	–	–
Drug-susceptible/Treatment naïve and MDR TB					
Video-observed therapy	Directly observed treatment	**12 Comparisons in Nsengiyumva (2018) [Brazil-LMIC]** Cost-effectiveness was estimated according to patient drug resistance (drug-susceptible TB cohort and MDR-TB cohort) and perspectives (health system perspective and societal perspective). All interventions led to cost savings compared with standard, directly observed treatment.	100% (4/4)	–	–
Medication monitor (a small device attached to standard pill dispenser)			100% (4/4)	–	–
Medication monitor (Taking pills from blister pack daily reveals random toll-free numbers)			100% (4/4)	–	–

MDR-TB, multidrug-resistant tuberculosis; DOT, directly observed treatment; LMIC, low- and middle-income country; HIC, high-income country.

Results of the cost-effectiveness analysis are presented in Supplementary Table S2 for each study.

### 3.5 Quality Assessment

The results of the quality assessment are presented in Supplementary Table S3. In general, most items were fulfilled in the selected 17 articles, but data was not available for all 17 studies for Q4, the criterion regarding pre-specified subgroup analysis. For Q16, the criteria regarding the disclosure of funding, all the included articles except for one (Schnippel et al., 2018b) disclosed the source of funding. Five studies were funded by the pharmaceutical industry. For all five studies, the intervention, including bedaquiline, was more cost-effective than the comparators (Wolfson et al., 2015; Park et al., 2016; Codecasa et al., 2017; Lu et al., 2017; Wirth et al., 2017). Three (Schnippel et al., 2018a; John et al., 2018; Fan et al., 2019) declared no funding, and one (Schnippel et al., 2018b) did not clarify whether it had been funded.

Some studies did not fulfill all of the criteria of the QHES instrument. Manabe et al. (2012) did not conduct an incremental analysis between alternatives (Q6) or indicate the analytic horizon and discount rate (Q8), rating among the lowest of the included 17 studies. Another study by Loveday et al. (2018) also rated among the lowest because it did not explain the economic model of the research, receiving a “No” for Q12, 13, the criteria regarding the economic model of the study. The study by Schnippel et al. (2018b) had the second-lowest scores because it did not clearly disclose the funding source, receiving a “No” for Q16. Hunchangsith et al. (2012) did not state the analytic horizon and discount rate; hence, the study was rated as “partial” for Q8. The remaining studies fulfilled all the QHES items, except for Q4.

## 4 Discussion

We found that there have been several economic evaluations that suggest a shortened duration of the anti-TB regimen from 6-month to 4 months or 2-month, and novel regimens with new combinations of existing drugs (e.g., ethambutol, moxifloxacin) or new drugs (e.g., bedaquiline). In terms of care, decentralized care (home-based or mobile device-based) and DOT have been suggested against existing hospital-based centralized care and self-administered treatment mainly in LMICs. The studies we examined generally concluded that novel regimens with new medications or combinations, shorter regimens, and more decentralized care appeared more cost-effective than traditional anti-TB interventions.

Given that the standard 6-month regimen for TB treatment has faced challenges regarding patient adherence, shortened regimens (e.g., 2 or 4 months) have been suggested. Shortened regimens have the advantage of reducing the burden on patients and health systems. Moreover, patients have additional benefits, such as shortened length of interrupted daily life, a shorter period of side effects, and better adherence outcomes (Gospodarevskaya et al., 2014). Although a randomized clinical trial concluded that noninferiority of 4-month regimens with moxifloxacin to the standard 6-month regimen was not proven, bacterial loads decreased more rapidly with the shortened regimen than with the standard regimen (Gillespie et al., 2014). Along with the expectations of a shortened TB regimen to enhance treatment adherence and outcomes, our study revealed that in most cases, shortened regimens are more cost-effective than the standard 6-month regimen. However, one study (Gomez et al., 2016) concluded that a 4-month regimen was not more cost-effective than the standard 6-month regimen in Bangladesh because the ICER exceeded the willingness-to-pay threshold of Bangladesh, which was based on one gross domestic product per capita. In that study, cost reduction from shortened duration was the lowest, and increased health service costs from new regimens were the largest with a 60% increase rate in Bangladesh compared with other countries (South Africa, Brazil, and Tanzania), resulting in a trade-off between the new regimen’s high prices and low service delivery costs. Considering 3 out of 4 studies which assessed shortened regimen were conducted in LMICs, the need for shortened regimen to enhance patients’ treatment adherence was more noticeable in LMICs compared to HICs.

There have been concerns regarding low TB treatment adherence and outcomes in LMICs, arguing inaccessibility to treatment center, rural residence, lack of family support, and level of education to be risk factors (Ali and Prins, 2016; Ali et al., 2019). Therefore, care strategies to improve treatment adherence have been developed and assessed in the region. For example, decentralized TB care has more treatment success than centralized (hospital-based) TB care and improved treatment adherence, leading to lower health system costs, especially in LMICs such as Nigeria and South Africa (Ho et al., 2017). Likewise, in our review, treatment strategies to improve treatment adherence such as shortened regimens and care strategies were mainly assessed as cost-effective in LMICs. The care strategies included decentralized community-based care (Loveday et al., 2018), home-based care (John et al., 2018), and device-aided care (Nsengiyumva et al., 2018) which could aid patients with inaccessibility in LMICs. Cost-effectiveness results with treatment outcomes from prospective cohort study (Loveday et al., 2018) and randomized clinical trial (John et al., 2018) both concluded that decentralized care strategies are more cost-effective than centralized care.

Of the LMICs, TB patients in sub-Saharan Africa are particularly suffering from concomitant HIV, with more than 50% of TB patients co-infected with TB and HIV in the region (WHO, 2019). Co-infection with TB and HIV is a risk factor for low TB treatment adherence because medications for two diseases would result in a lot of diverse pills and their adverse events (Muture et al., 2011; Garrido Mda et al., 2012; Tola et al., 2015). Therefore, HIV status should be considered in economic evaluations for TB treatment in sub-Saharan Africa. In our study, we could identify how HIV status was reflected in various economic evaluation models of the region. Six studies used several economic evaluation models to reflect HIV or ART status, such as Markov model, decision tree model, transmission model, individual-based decision analytic model, and prospective cohort design. The cost-effectiveness results differed by HIV or ART status. Cost-effectiveness was improved when the proportion of HIV-positive patients was lower (Schnippel et al., 2018a), and when survival in ART was higher (Gomez et al., 2016).

Though HICs bear only 5% of the global TB burden, HICs have long been interested in MDR-TB treatments (Bastian et al., 2003). Most studies which showed evidence of successful management in MDR-TB treatments have been conducted in HICs (Mukherjee et al., 2004). This might have stemmed from plentiful resources such as accurate drug susceptibility test or funds for expensive therapy in HICs (Seaworth, 2002). The attention to MDR-TB treatment in HICs was also seen in our study. Six out of 8 studies which were conducted in HICs were assessing new regimen (e.g., bedaquiline-based regimen) for MDR-TB. Considering new treatment strategies such as individualized treatment for MDR-TB are getting more attention in HICs (Chang et al., 2021), future economic evaluation studies will be needed to assess new strategies for MDT-TB.

A new regimen with diarylquinoline (e.g., bedaquiline) leads to faster culture conversion (Diacon et al., 2014). Likewise, we confirmed that the bedaquiline plus background regimen was more cost-effective than the background regimen for MDR-TB patients who failed the background regimen or for XDR-TB patients. In addition, bedaquiline-based regimens were more cost-effective than regimens containing delamanid or linezolid for MDR-TB patients. Compared with the background regimen alone, adding bedaquiline to the background regimen was more cost-effective, except when adverse drug reactions were not considered (Schnippel et al., 2018b). Considering that 83% of patients under drug-resistant TB treatment experience adverse drug reaction (Schnippel et al., 2017), it would be justifiable to consider adverse drug reactions when assessing the cost-effectiveness of the regimens for MDR-TB patients. In the included studies in our study, the adverse reaction of bedaquiline was reflected in economic evaluation models either by including cost for screening adverse reaction (Schnippel et al., 2018a) or cost for treating adverse reaction with detailed frequency of occurrence (Wirth et al., 2017). Moreover, considering most included comparisons were conducted with treatment outcome data earned from randomized clinical trials, the safety/tolerability data in randomized clinical trials were reflected in parameters of economic evaluations. The most frequently used outcomes, DALYs and QALYs also imply patients’ quality of life which reflect patients’ discomfort from safety and tolerability issues. Therefore, the cost-effectiveness results of the bedaquiline-based regimens upon the background regimen would be applicable to actual circumstances in which safety issues should be considered.

Throughout this review, DALYs, which present the impact of the intervention on patients’ lives, were most frequently used as a primary outcome (10 out of 17), followed by QALYs. Tuberculosis is one of the leading causes of global DALYs, rating 12th among 369 diseases and injuries in 2019 (Vos et al., 2020). Given that authoritative bodies, such as the WHO, encourage the use of DALYs when comparing the burden of disease across countries (Salomon et al., 2012), DALYs were used as a primary outcome for economic evaluations of diseases in which patient disability is mainly considered. For example, a previous systematic review on economic evaluations of pneumococcal vaccination revealed that DALYs were the most frequently used outcome along with QALYs (Saokaew et al., 2016). As DALYs are frequently used in economic evaluations for some diseases, the WHO’s Choosing Interventions that are Cost-Effective project suggested a one to three times the average per capita income for a given country or region as a general willingness-to-pay threshold for DALYs (Hutubessy et al., 2003). Seven of the 17 included articles mentioned about one to three times the gross domestic product threshold standards for DALYs (Owens et al., 2013; Knight et al., 2015; Gomez et al., 2016; Lu et al., 2017; Schnippel et al., 2018a; John et al., 2018; Fan et al., 2019). Owens et al. (2013) mentioned the threshold as one to three times the gross domestic product but omitted the exact value, making it difficult to define cost-effectiveness with estimated ICERs. Likewise, the standard used to define cost-effectiveness in the analysis is often missing. In our study, five studies (Manabe et al., 2012; Law et al., 2014; Schnippel et al., 2018b; Loveday et al., 2018; Nsengiyumva et al., 2018) omitted to state thresholds for a given outcome, and four studies (Wolfson et al., 2015; Park et al., 2016; Lu et al., 2017) suggested thresholds for only part of some of the given outcomes. For these cases, we had to evaluate the cost-effectiveness of the interventions using authors’ statements unless the intervention was dominant, which meant that it was more effective and less costly than the comparator. Therefore, there could be uncertainties in the cost-effectiveness of studies that did not suggest exact thresholds.

Most published economic evaluations tend to have favorable results for interventions, increasing the risk of bias. A previous systematic review of published cost-effectiveness analysis (Bell et al., 2006) revealed that about half of the economic evaluations reported ICERs below $20,000/QALY. In addition, it was shown in another systematic review that industry-funded studies report more favorable ICER than non-industry-funded studies (Bilcke et al., 2018). The results of these previous reviews demonstrated the possibility of publication or sponsorship bias in published economic evaluation studies. In our study, among the eight studies that concluded that bedaquiline-based regimens were cost-effective compared with background regimens, five (Wolfson et al., 2015; Park et al., 2016; Codecasa et al., 2017; Lu et al., 2017; Wirth et al., 2017) were funded by pharmaceutical companies, which may have introduced sponsorship bias. However, three studies (Schnippel et al., 2018a; Schnippel et al., 2018b; Fan et al., 2019) found bedaquiline-based regimens to be cost-effective than the standard regimens even without funding. Moreover, because there were articles that concluded interventions, such as a 4-month regimen (Gomez et al., 2016), an ethambutol-added regimen (Law et al., 2014), or a bedaquiline-based regimen (Schnippel et al., 2018b), which were generally more cost-effective than the standard regimen in most comparisons, were not cost-effective, our study showed favorable and unfavorable results simultaneously.

Our study is the first to conduct a systematic review of economic evaluation of treatments for active TB. Unlike some previous systematic reviews that did not assess the quality of articles (Nienhaus et al., 2011; Verdier et al., 2011; Marks et al., 2017), we carried out a quality assessment using the QHES instrument, which has been validated by clinicians and health economists. Moreover, by organizing the cost-effectiveness of each comparison of regimens and care strategies, we enabled the identification of frequently suggested interventions and their cost-effectiveness in published articles. In addition, the factors or settings that made interventions not cost-effective were determined with a detailed explanation of interventions that were not cost-effective or unavailable to assess in each comparison. The key components of recent economic evaluations for active TB that we arranged would help future studies set up an economic model for economic evaluations in active TB treatments.

Our study might have potential limitations. First, to cope with low treatment adherence in TB treatment, treatment strategies such as fixed-dose combinations of two or more anti-TB medications have been developed to simplify complexity in taking medication. Previous studies compared treatment outcomes, efficacy, or safety of fixed-dose combinations and single-drug formulations (Albanna et al., 2013; Gallardo et al., 2016; Lima et al., 2017), but economic evaluation study for fixed-dose combinations was scarce. As a result, there was no economic evaluation study of fixed-dose combinations for TB treatment that fulfilled our inclusion criteria. Economic evaluations for such treatment adherence-enhancing strategies will need to be reviewed afterward. Second, through a systematic review, organized estimates of each study could have been pooled using a meta-analysis method. However, there were heterogeneities between characteristics of studies in terms of study setting, population, methodology, and type of outcomes. For example, primary outcomes varied with the forms of DALYs, QALYs, LYG, TB case, death, treatment success rate, acquired resistance, cost savings, and mortality rate. To show the unique characteristics of each study, we did not compromise the results into one estimate, but listed features of each separate study by classifying the information according to patient characteristics (treatment naïve or MDR-TB) and type of interventions (drug or care). Therefore, we believe our study summarized the characteristics of recent studies, reflecting the patient and intervention, thereby minimizing the bias from heterogeneities.

## 5 Conclusion

Economic evaluation studies of past 10 years have suggested new strategies to improve therapeutic outcomes by enhancing treatment adherence in active TB treatment. New regimens with shorter durations (2 or 4 months) and decentralized care have been suggested as cost-effective mainly in LMICs. Novel regimens with new anti-TB agents (e.g., bedaquiline) have been suggested as cost-effective. This review provides information on the economic evaluation of treatments for active TB, which can help future research on the topic set up economic evaluation model.

## Data Availability

The original contributions presented in the study are included in the article/Supplementary Material, further inquiries can be directed to the corresponding authors.

## Abbreviations

ART, antiretroviral therapy; BR, background regimen; DALY, disability-adjusted life years; DOT, directly observed treatment; HIC, high-income country; HIV, human immunodeficiency virus; ICER, incremental cost-effectiveness ratio; LMIC, low- and middle-income country; MDR-TB, multidrug-resistant tuberculosis; QALY, quality-adjusted life years; QHES, quality of health economic studies; TB, tuberculosis; XDR-TB, extremely drug-resistant tuberculosis
