# Supplementary material for: A Systematic Review of Economic Evaluations of Active Tuberculosis Treatments
Source: Front Pharmacol. 2021 Dec 13;12:736986. doi: 10.3389/fphar.2021.736986 (PMC8710595; doi:10.3389/fphar.2021.736986)
Supplement: Supplementary file 1 [file DataSheet1.docx]

Supplementary Material

**Supplemental Table 1. Search strategies of the different databases**

| **Database** | **Search Terms** |
| --- | --- |
| EMBASE |  |
| #1 | 'tuberculosis'/exp OR tuberculosis:ab,ti |
| #2 | 'economic evaluation'/exp |
| #3 | cost:ab,ti AND effective:ab,ti |
| #4 | cost:ab,ti AND utilit*:ab,ti |
| #5 | #2 OR #3 OR #4 |
| #6 | 'economic model'/exp OR model*:ab,ti |
| #7 | #1 AND #5 AND #6 |
| Pubmed |  |
| #1 | “Tuberculosis”[tiab] OR “Tuberculosis” [MeSH] |
| #2 | “Cost-Benefit Analysis” [Mesh] OR (cost[tiab] AND effective*[tiab]) OR (cost[tiab] AND utility[tiab]) |
| #3 | model*[tiab] OR model, economic [MeSH] |
| #4 | #1 AND#2 AND #3 |
| Cochrane |  |
| #1 | MeSH descriptor: [Tuberculosis] explode all trees |
| #2 | (Tuberculosis*):ti,ab |
| #3 | #1 OR #2 |
| #4 | MeSH descriptor: [Cost-Benefit Analysis] explode all trees |
| #5 | (cost AND effective*):ti,ab |
| #6 | (cost and utilit*):ti,ab |
| #7 | #4 OR #5 OR #6 |
| #8 | MeSH descriptor: [Models, Economic] explode all trees |
| #9 | (model*):ti,ab |
| #10 | #8 OR #9 |
| #11 | #3 AND #7 AND #10 |

exp, explosion; ab, abstract; ti, title; tiab, title/abstract; MeSH, medical subject heading

Supplemental Table 2. Results of economic evaluations of the included 17 studies

| **Study** | **Intervention** | **Comparator** | **Outcome** | | **Threshold of cost-effectiveness**  **(USD)** | **Cost-effectiveness** | **Note** | **Funding** | **Source of Funding** |
| --- | --- | --- | --- | --- | --- | --- | --- | --- | --- |
|  |  |  | **Type** | **Values (USD)** |  |  |  |  |  |
| Codecasa (2017) | Bedaquiline + BR | BR | ICER (Cost/LYG) | 18,667 | between 28,047 and 67,312 | Y | NHS perspective | Y | Janssen-Cilag |
| Codecasa (2017) | Bedaquiline + BR | BR | ICER (Cost/LYG) | 4,578 |  | Y | Societal perspective |  |  |
| Fan (2019) | Bedaquiline + BR | BR | ICUR (Cost/QALY) | 12 | 46,182 | Y |  | N | No funding |
| Fan (2019) | Delamanid + BR | BR | ICUR (Cost/QALY) | 1,680,333 |  | N |  |  |  |
| Gomez (2016) | 4-month regimen (new regimen) | 6-month regimen (standard) | ICER (Cost/DALY) | Cost saving | 6618 | Y | South Africa, guidelines adhered DALYS averted of intervention were larger than that of the comparator in all settings | Y | Global Alliance for TB Drug Development RGW funded by the Medical Research Council (UK) (MR/J005088/1), the Bill and Melinda Gates Foundation (TB Modelling and Analysis Consortium: OPP1084276), and CDC/PEPFAR via the Aurum Institute |
| Gomez (2016) | 4-month regimen (new regimen) | 6-month regimen (standard) | ICER (Cost/DALY) | Cost saving | 11,208 | Y | Brazil, guidelines adhered DALYS averted of intervention were larger than that of the comparator in all settings |  |  |
| Gomez (2016) | 4-month regimen (new regimen) | 6-month regimen (standard) | ICER (Cost/DALY) | 1472 | 829 | N | Bangladesh, guidelines adhered DALYS averted of intervention were larger than that of the comparator in all settings |  |  |
| Gomez (2016) | 4-month regimen (new regimen) | 6-month regimen (standard) | ICER (Cost/DALY) | Cost saving | 695 | Y | Tanzania, guidelines adhered  DALYS averted of intervention were larger than that of the comparator in all settings |  |  |
| Gomez (2016) | 4-month regimen (new regimen) | 6-month regimen (standard) | ICER (Cost/DALY) | 13.6 | 6618 | Y | South Africa, the current scenario DALYS averted of intervention were larger than that of the comparator in all settings |  |  |
| Gomez (2016) | 4-month regimen (new regimen) | 6-month regimen (standard) | ICER (Cost/DALY) | Cost saving | 11,208 | Y | Brazil, the current scenario DALYS averted of intervention were larger than that of the comparator in all settings |  |  |
| Gomez (2016) | 4-month regimen (new regimen) | 6-month regimen (standard) | ICER (Cost/DALY) | 1220 | 829 | N | Bangladesh, the current scenario DALYS averted of intervention were larger than that of the comparator in all settings |  |  |
| Gomez (2016) | 4-month regimen (new regimen) | 6-month regimen (standard) | ICER (Cost/DALY) | 161 | 695 | Y | Tanzania, the current scenario DALYS averted of intervention were larger than that of the comparator in all settings |  |  |
| Hunchangsith (2012) | DOT by an HW | SAT (self-administered treatment) | ICER (Cost/DALY) | 1100 | 9000 | NA | For all interventions, cost-effectiveness could not be decided due to wide uncertainty ranges that crossed zero | Y | Welcome Trust, UK National Health and Medical Research Council of Australia |
| Hunchangsith (2012) | DOT by a community member | SAT (self-administered treatment) | ICER (Cost/DALY) | Dominant |  | NA | For all interventions, cost-effectiveness could not be decided due to wide uncertainty ranges that crossed zero |  |  |
| Hunchangsith (2012) | DOT by a family member | SAT (self-administered treatment) | ICER (Cost/DALY) | Dominant |  | NA | For all interventions, cost-effectiveness could not be decided due to wide uncertainty ranges that crossed zero |  |  |
| Hunchangsith (2012) | Mobile phone | SAT (self-administered treatment) | ICER (Cost/DALY) | 350 |  | NA | For all interventions, cost-effectiveness could not be decided due to wide uncertainty ranges that crossed zero |  |  |
| John (2018) | Decentralized MDR-TB treatment (home-based) | Centralized treatment (hospital-based) | ICUR (Cost/QALY) | 2383 | 5584.5 | Y |  | N | No funding |
| Knight (2015) | 4-month regimen (new regimen) | 6-month regimen (standard) | Cost-effective cost/month of 4-month regimen | 436 | 6618 | Y |  | Y | Global Alliance for TB Drug Development, New York, USA RGW is funded by the Medical Research Council (UK) and the Bill and Melinda Gates Foundation |
| Law (2014) | Ethambutol added to initial treatment | Standard treatment | ICER (Cost/DALY) | Less effective | NR | N | 5% INH monoresistant TB, 1% MDR-TB | Y | Canadian Institutes of Health Research |
| Law (2014) | Strengthened retreatment | Standard treatment | ICER (Cost/DALY) | Dominant | NR | Y | 5% INH monoresistant TB, 1% MDR-TB |  |  |
| Law (2014) | Standardized MDR treatment for failures of initial treatment | Standard treatment | ICER (Cost/DALY) | 5745 | NR | Y | 5% INH monoresistant TB, 1% MDR-TB |  |  |
| Law (2014) | Ethambutol added to initial treatment | Standard treatment | ICER (Cost/DALY) | Less effective | NR | N | 15% INH monoresistant TB, 1% MDR-TB |  |  |
| Law (2014) | Strengthened retreatment | Standard treatment | ICER (Cost/DALY) | Dominant | NR | Y | 15% INH monoresistant TB, 1% MDR-TB |  |  |
| Law (2014) | Standardized MDR treatment for failures of initial treatment | Standard treatment | ICER (Cost/DALY) | 4867 | NR | Y | 15% INH monoresistant TB, 1% MDR-TB |  |  |
| Law (2014) | Ethambutol added to initial treatment | Standard treatment | ICER (Cost/DALY) | Less effective | NR | N | 5% INH monoresistant TB, 10% MDR-TB |  |  |
| Law (2014) | Strengthened retreatment | Standard treatment | ICER (Cost/DALY) | Dominant | NR | Y | 5% INH monoresistant TB, 10% MDR-TB |  |  |
| Law (2014) | Standardized MDR treatment for failures of initial treatment | Standard treatment | ICER (Cost/DALY) | 2857 | NR | Y | 5% INH monoresistant TB, 10% MDR-TB |  |  |
| Law (2014) | Ethambutol added to initial treatment | Standard treatment | ICER (Cost/DALY) | Less effective | NR | N | 15% INH monoresistant TB, 10% MDR-TB |  |  |
| Law (2014) | Strengthened retreatment | Standard treatment | ICER (Cost/DALY) | Dominant | NR | Y | 15% INH monoresistant TB, 10% MDR-TB |  |  |
| Law (2014) | Standardized MDR treatment for failures of initial treatment | Standard treatment | ICER (Cost/DALY) | 2860 | NR | Y | 15% INH monoresistant TB, 10% MDR-TB |  |  |
| Law (2014) | Ethambutol added to initial treatment | Standard treatment | ICER (Cost/MDR case) | Dominant | NR | Y | 5% INH monoresistant TB, 1% MDR-TB |  |  |
| Law (2014) | Strengthened retreatment | Standard treatment | ICER (Cost/MDR case) | Dominant | NR | Y | 5% INH monoresistant TB, 1% MDR-TB |  |  |
| Law (2014) | Standardized MDR treatment for failures of initial treatment | standard treatment | ICER (Cost/MDR case) | 58,6978 | NR | NA | 5% INH monoresistant TB, 1% MDR-TB |  |  |
| Law (2014) | Ethambutol added to initial treatment | Standard treatment | ICER (Cost/MDR case) | Dominant | NR | Y | 15% INH monoresistant TB, 1% MDR-TB |  |  |
| Law (2014) | Strengthened retreatment | Standard treatment | ICER (Cost/MDR case) | Dominant | NR | Y | 15% INH monoresistant TB, 1% MDR-TB |  |  |
| Law (2014) | Standardized MDR treatment for failures of initial treatment | Standard treatment | ICER (Cost/MDR case) | 721,168 | NR | NA | 15% INH monoresistant TB, 1% MDR-TB |  |  |
| Law (2014) | Ethambutol added to initial treatment | Standard treatment | ICER (Cost/MDR case) | Dominant | NR | Y | 5% INH monoresistant TB, 10% MDR-TB |  |  |
| Law (2014) | Strengthened retreatment | Standard treatment | ICER (Cost/MDR case) | Dominant | NR | Y | 5% INH monoresistant TB, 10% MDR-TB |  |  |
| Law (2014) | Standardized MDR treatment for failures of initial treatment | Standard treatment | ICER (Cost/MDR case) | 2,958,162 | NR | NA | 5% INH monoresistant TB, 10% MDR-TB |  |  |
| Law (2014) | Ethambutol added to initial treatment | Standard treatment | ICER (Cost/MDR case) | Dominant | NR | Y | 15% INH monoresistant TB, 10% MDR-TB |  |  |
| Law (2014) | Strengthened retreatment | Standard treatment | ICER (Cost/MDR case) | Dominant | NR | Y | 15% INH monoresistant TB, 10% MDR-TB |  |  |
| Law (2014) | Standardized MDR treatment for failures of initial treatment | Standard treatment | ICER (Cost/MDR case) | 2,599,325 | NR | NA | 15% INH monoresistant TB, 10% MDR-TB |  |  |
| Law (2014) | Ethambutol added to initial treatment | Standard treatment | ICER (Cost/TB death) | Dominant | NR | Y | 5% INH monoresistant TB, 1% MDR-TB |  |  |
| Law (2014) | Strengthened retreatment | Standard treatment | ICER (Cost/TB death) | Dominant | NR | Y | 5% INH monoresistant TB, 1% MDR-TB |  |  |
| Law (2014) | Standardized MDR treatment for failures of initial treatment | Standard treatment | ICER (Cost/TB death) | 46,958 | NR | NA | 5% INH monoresistant TB, 1% MDR-TB |  |  |
| Law (2014) | Ethambutol added to initial treatment | Standard treatment | ICER (Cost/TB death) | Dominant | NR | Y | 15% INH monoresistant TB, 1% MDR-TB |  |  |
| Law (2014) | Strengthened retreatment | Standard treatment | ICER (Cost/TB death) | Dominant | NR | Y | 15% INH monoresistant TB, 1% MDR-TB |  |  |
| Law (2014) | Standardized MDR treatment for failures of initial treatment | standard treatment | ICER (Cost/TB death) | 39,697 | NR | NA | 15% INH monoresistant TB, 1% MDR-TB |  |  |
| Law (2014) | Ethambutol added to initial treatment | Standard treatment | ICER (Cost/TB death) | Dominant | NR | Y | 5% INH monoresistant TB, 10% MDR-TB |  |  |
| Law (2014) | Strengthened retreatment | Standard treatment | ICER (Cost/TB death) | Dominant | NR | Y | 5% INH monoresistant TB, 10% MDR-TB |  |  |
| Law (2014) | Standardized MDR treatment for failures of initial treatment | Standard treatment | ICER (Cost/TB death) | 23,477 | NR | NA | 5% INH monoresistant TB, 10% MDR-TB |  |  |
| Law (2014) | Ethambutol added to initial treatment | Standard treatment | ICER (Cost/TB death) | Dominant | NR | Y | 15% INH monoresistant TB, 10% MDR-TB |  |  |
| Law (2014) | Strengthened retreatment | Standard treatment | ICER (Cost/TB death) | Dominant | NR | Y | 15% INH monoresistant TB, 10% MDR-TB |  |  |
| Law (2014) | Standardized MDR treatment for failures of initial treatment | Standard treatment | ICER (Cost/TB death) | 23,488 | NR | NA | 15% INH monoresistant TB, 10% MDR-TB |  |  |
| Loveday (2018) | Decentralized (hospitalization for whole injectable phase) | Centralized hospital | ICER (Cost/treatment success rate) | -179 | NR | Y | Success rate increased but cost decreased in decentralized compared with centralized | Y | The South African Medical Research Council, Izumi Foundation, University Research Co-operation (URC) United Way Worldwide grant |
| Loveday (2018) | Community- based / clinic | Decentralized(hospitalization for whole injectable phase) | ICER (Cost/treatment success rate) | -2738 | NR | Y | Success rate increased but cost decreased in clinic compared with decentralized |  |  |
| Loveday (2018) | Community- based / mobile | Community- based / clinic | ICER (Cost/treatment success rate) | 402 | NR | Y | ICER was justifiable (indicating that mobile model was more cost-effective) |  |  |
| Loveday (2018) | Community- based / mobile | Decentralized (initial hospitalization for all patients) | ICER (Cost/treatment success rate) | 9687 | NR | Y | ICER was not justifiable (indicating that decentralized care was not more cost-effective) Mobile model was the most cost-effective model |  |  |
| Lu (2017) | Bedaquiline + BR | BR | incremental change in DALYs | -20.90% | 50532 | Y | Estonia Threshold unit: cost/DALY averted | Y | Janssen Pharmaceuticals |
| Lu (2017) | Bedaquiline + BR | BR | incremental change in DALYs | -19.51% | 42111 | Y | Russia Threshold unit: cost/DALY averted |  |  |
| Lu (2017) | Bedaquiline + BR | BR | incremental change in DALYs | -18.35% | 22056 | Y | South Africa Threshold unit: cost/DALY averted |  |  |
| Lu (2017) | Bedaquiline + BR | BR | Incremental change in DALYs | -20.59% | 20388 | Y | Peru Threshold unit: cost/DALY averted |  |  |
| Lu (2017) | Bedaquiline + BR | BR | Incremental change in DALYs | -25.15% | 18273 | Y | China Threshold unit: cost/DALY averted |  |  |
| Lu (2017) | Bedaquiline + BR | BR | Incremental change in DALYs | -14.16% | 7761 | Y | Philippines Threshold unit: cost/DALY averted |  |  |
| Lu (2017) | Bedaquiline + BR | BR | Incremental change in DALYs | -22.18% | 4509 | Y | India Threshold unit: cost/DALY averted |  |  |
| Lu (2017) | Bedaquiline + BR | BR | Incremental change in successful outcomes | 54.67% | NR | Y | Estonia |  |  |
| Lu (2017) | Bedaquiline + BR | BR | Incremental change in successful outcomes | 55.78% | NR | Y | Russia |  |  |
| Lu (2017) | Bedaquiline + BR | BR | Incremental change in successful outcomes | 55.02% | NR | Y | South Africa |  |  |
| Lu (2017) | Bedaquiline + BR | BR | Incremental change in successful outcomes | 54.87% | NR | Y | Peru |  |  |
| Lu (2017) | Bedaquiline + BR | BR | Incremental change in successful outcomes | 51.62% | NR | Y | China |  |  |
| Lu (2017) | Bedaquiline + BR | BR | incremental change in successful outcomes | 60.78% | NR | Y | Philippines |  |  |
| Lu (2017) | Bedaquiline + BR | BR | Incremental change in successful outcomes | 52.87% | NR | Y | India |  |  |
| Lu (2017) | Bedaquiline + BR | BR | Incremental changes in acquired resistance | -31.59% | NR | Y | Estonia |  |  |
| Lu (2017) | Bedaquiline + BR | BR | Incremental changes in acquired resistance | -31.75% | NR | Y | Russia |  |  |
| Lu (2017) | Bedaquiline + BR | BR | Incremental changes in acquired resistance | -40.80% | NR | Y | South Africa |  |  |
| Lu (2017) | Bedaquiline + BR | BR | Incremental changes in acquired resistance | -31.62% | NR | Y | Peru |  |  |
| Lu (2017) | Bedaquiline + BR | BR | Incremental changes in acquired resistance | -31.34% | NR | Y | China |  |  |
| Lu (2017) | Bedaquiline + BR | BR | Incremental changes in acquired resistance | -32.22% | NR | Y | Philippines |  |  |
| Lu (2017) | Bedaquiline + BR | BR | Incremental changes in acquired resistance | -31.61% | NR | Y | India |  |  |
| Manabe (2012) | 6-month regimen with isoniazid and rifampicin for 4 months (4HR) in continuation treatment | 8-month regimen with isoniazid and ethambutol for 6 months (6HE) in continuation treatment | Cost savings per person | 2.42 | NR | Y | ICER not calculated since 4HR was a dominant strategy | Y | Infectious Disease Institute |
| Manabe (2012) | 6-month regimen with isoniazid and rifampicin for 4 months (4HR) in continuation treatment | 8-month regimen with isoniazid and ethambutol for 6 months (6HE) in continuation treatment | Mortality rate reduction | 4.50% | NR | Y | ICER not calculated since 4HR was a dominant strategy |  |  |
| Nsengiyumva (2018) | VOT (Video-observed therapy) | DOT: directly observed treatment | Incremental savings | 363 | NR | Y | Drug-susceptible TB cohort - Health system perspective | Y | European Respiratory Society |
| Nsengiyumva (2018) | VOT (Video-observed therapy) | DOT: directly observed treatment | Incremental savings | 531 | NR | Y | Drug-susceptible TB cohort - Societal perspective |  |  |
| Nsengiyumva (2018) | VOT (Video-observed therapy) | DOT: directly observed treatment | Incremental savings | 1409 | NR | Y | MDR-TB cohort - Health system perspective |  |  |
| Nsengiyumva (2018) | VOT (Video-observed therapy) | DOT: directly observed treatment | Incremental savings | 2002 | NR | Y | MDR-TB cohort - Societal perspective |  |  |
| Nsengiyumva (2018) | MM (Medication monitor)-Wisepill | DOT: directly observed treatment | Incremental savings | 507 | NR | Y | Drug-susceptible TB cohort - Health system perspective |  |  |
| Nsengiyumva (2018) | MM (Medication monitor)-Wisepill | DOT: directly observed treatment | Incremental savings | 675 | NR | Y | Drug-susceptible TB cohort - Societal perspective |  |  |
| Nsengiyumva (2018) | MM (Medication monitor)-Wisepill | DOT: directly observed treatment | Incremental savings | 1831 | NR | Y | MDR-TB cohort - Health system perspective |  |  |
| Nsengiyumva (2018) | MM (Medication monitor)-Wisepill | DOT: directly observed treatment | Incremental savings | 2425 | NR | Y | MDR-TB cohort - Societal perspective |  |  |
| Nsengiyumva (2018) | MM (Medication monitor)-99DOTS | DOT: directly observed treatment | Incremental savings | 536 | NR | Y | Drug-susceptible TB cohort - Health system perspective |  |  |
| Nsengiyumva (2018) | MM (Medication monitor)-99DOTS | DOT: directly observed treatment | Incremental savings | 704 | NR | Y | Drug-susceptible TB cohort - Societal perspective |  |  |
| Nsengiyumva (2018) | MM (Medication monitor)-99DOTS | DOT: directly observed treatment | Incremental savings | 1870 | NR | Y | MDR-TB cohort - Health system perspective |  |  |
| Nsengiyumva (2018) | MM (Medication monitor)-99DOTS | DOT: directly observed treatment | Incremental savings | 2463 | NR | Y | MDR-TB cohort - Societal perspective |  |  |
| Owens (2013) | Novel treatment involving moxifloxacin-2 months | Standard treatment – 6 months | ICER (Cost/DALY) | 1400 | 1-3 X GDP | NA | Low treatment cost For all comparisons, 2- or 4-month regimen was more effective compared with the standard 6-month regimen. The threshold was mentioned as 1-3 times GDP, but the exact amount was not mentioned clearly. | Y | US National Institutes of Health |
| Owens (2013) | Novel treatment involving moxifloxacin-2 months | Standard treatment - 6 months | ICER (Cost/DALY) | 1000 |  | NA | Moderate treatment cost For all comparisons, 2- or 4-month regimen was more effective compared with the standard 6-month regimen. The threshold was mentioned as 1-3 times GDP, but the exact amount was not mentioned clearly. |  |  |
| Owens (2013) | Novel treatment involving moxifloxacin-2 months | Standard treatment - 6 months | ICER (Cost/DALY) | Preferred |  | Y | High treatment cost less costly and more effective For all comparisons, 2- or 4-month regimen was more effective compared with the standard 6-month regimen. The threshold was mentioned as 1-3 times GDP, but the exact amount was not mentioned clearly. |  |  |
| Owens (2013) | Novel treatment involving moxifloxacin-4 months | Standard treatment - 6 months | ICER (Cost/DALY) | 740 |  | Y | Low treatment cost For all comparisons, 2- or 4-month regimen was more effective compared with the standard 6-month regimen. The threshold was mentioned as 1-3 times GDP, but the exact amount was not mentioned clearly. A 4-month first-line regimen would be cost-effective in all high-burden countries using 1-3 GDP threshold. |  |  |
| Owens (2013) | Novel treatment involving moxifloxacin-4 months | Standard treatment - 6 months | ICER (Cost/DALY) | 430 |  | Y | Moderate treatment cost For all comparisons, 2- or 4-month regimen was more effective compared with the standard 6-month regimen. The threshold was mentioned as 1-3 times GDP, but the exact amount was not mentioned clearly. A 4-month first-line regimen would be cost-effective in all high-burden countries using 1-3 GDP threshold. |  |  |
| Owens (2013) | Novel treatment involving moxifloxacin-4 months | Standard treatment - 6 months | ICER (Cost/DALY) | Preferred |  | Y | High treatment cost less costly and more effective For all comparisons, 2- or 4-month regimen was more effective compared with the standard 6-month regimen. The threshold was mentioned as 1-3 times GDP, but the exact amount was not mentioned clearly. A 4-month first-line regimen would be cost-effective in all high-burden countries using 1-3 GDP threshold. |  |  |
| Park (2016) | Bedaquiline + standard regimen (SR) | Standard regimen | ICER (Cost/LYG) | 9113 | NR | Y | Bedaquiline, as a part of combination therapy with SR, is a cost-effective option for the treatment of MDR-TB (including XDR-TB) compared with SR only | Y | Janssen Pharmaceuticals Korea |
| Park (2016) | Bedaquiline + standard regimen (SR) | Standard regimen | ICUR (Cost/QALY) | 9800 | 21892 | Y | Bedaquiline, as a part of combination therapy with SR, is a cost-effective option for the treatment of MDR-TB (including XDR-TB) compared with SR only |  |  |
| Schnippel (2018):1 | Bedaquiline-based regimen1 (capreomycin replaced with bedaquiline) | Standard regimen | ICER (Cost/DALY) | -3804 | 5718 | Y | Less cost and slightly more effectiveness (DALY averted) compared with the standard regimen threshold; 2015 per capita GDP for South Africa | N | No funding |
| Schnippel (2018):1 | Bedaquiline-based regimen2 (kanamycin replaced with bedaquiline) | Standard regimen | ICER (Cost/DALY) | 1242 | 5718 | Y | Threshold; 2015 per capita GDP for South Africa |  |  |
| Schnippel (2018):2 | Bedaquiline-based regimen | Injection-based regimen | ICER (Cost/DALY) | 516 | NR | N | No toxicity profile more costly and less effective | NR | NR |
| Schnippel (2018):2 | Bedaquiline-based regimen | Injection-based regimen | ICER (Cost/DALY) | Dominant | NR | Y | Adjusted for toxicity profile less costly and more effective | NR | NR |
| Wirth (2017) | Linezolid + BR | BR | ICUR (Cost/QALY) | 98,145 | Greater than 24681 | N | linezolid and delamanid were dominated by a combination of BR alone and BR plus bedaquiline | Y | Janssen Pharmaceutical Companies of Johnson and Johnson |
| Wirth (2017) | Delamanid + BR | BR | ICUR (Cost/QALY) | 43,420 |  | N | linezolid and delamanid were dominated by a combination of BR alone and BR plus bedaquiline |  |  |
| Wirth (2017) | Bedaquline + BR | BR | ICUR (Cost/QALY) | 24,948 |  | Y | bedaquline + BR is the most cost-effective treatment strategy |  |  |
| Wirth (2017) | Delamanid + BR | Linezolid + BR | ICUR (Cost/QALY) | 2273 |  | Y |  |  |  |
| Wirth (2017) | Bedaquline + BR | Linezolid + BR | ICUR (Cost/QALY) | 6492 |  | Y |  |  |  |
| Wirth (2017) | Bedaquline + BR | Delamanid + BR | ICUR (Cost/QALY) | 8721 |  | Y |  |  |  |
| Wolfson (2015) | Bedaquiline + BR | BR | ICER (Cost/DALY) | 699 | NR | Y | Dominant (less costly and more effective) | Y | Janssen Pharmaceutical Companies of Johnson and Johnson |
| Wolfson (2015) | Bedaquiline + BR | BR | ICUR (Cost/QALY) | 2792 | 5578 to 8368 | Y | Dominant (less costly and more effective) |  |  |

BR, background regimen; ICER, incremental cost-effectiveness ratio; ICUR, incremental cost-utility ratio; DALY, disability-adjusted life year; QALY, quality-adjusted life year; LYG, life year gained; Y, yes; N, no; NA, not available; NR, not reported.

The values of outcome and thresholds with currencies other than US dollars were converted into US dollars with exchange rates in July 2020.

Supplemental Table 3. Results of the QHES instrument for the 17 included studies

|  | **QHES criterion** | **Codecasa (2017)** | **Lu (2017)** | **Park (2016)** | **Schnippel (2018)(1)** | **Schnippel (2018)(2)** | **Fan (2019)** | **John (2018)** | **Loveday (2018)** | **Wirth (2017)** | **Wolfson (2015)** | **Gomez (2016)** | **Hunchangsith (2012)** | **Knight (2015)** | **Law (2014)** | **Manabe (2012)** | **Owens (2013)** | **Nsengiyumva (2018)** |
| --- | --- | --- | --- | --- | --- | --- | --- | --- | --- | --- | --- | --- | --- | --- | --- | --- | --- | --- |
| 1 | Was the study objective presented in a clear, specific, and measurable manner? | O | O | O | O | O | O | O | O | O | O | O | O | O | O | O | O | O |
| 2 | Were the perspective of the analysis (societal, third-party payer, etc.) and reasons for its selection stated? | O | O | O | O | O | O | O | O | O | O | O | O | O | O | O | O | O |
| 3 | Were variable estimates used in the analysis from the best available source (i.e., randomized control trial - best, expert opinion - worst)? | O | O | O | O | O | O | O | O | O | O | O | O | O | O | O | O | O |
| 4 | If estimates came from a subgroup analysis, were the groups prespecified at the beginning of the study? | NA | NA | NA | NA | NA | NA | NA | NA | NA | NA | NA | NA | NA | NA | NA | NA | NA |
| 5 | Was uncertainty handled by (1) statistical analysis to address random events, (2) sensitivity analysis to cover a range of assumptions? | O | O | O | O | O | O | O | O | O | O | O | O | O | O | O | O | O |
| 6 | Was incremental analysis performed between alternatives for resources and costs? | O | O | O | O | O | O | O | O | O | O | O | O | O | O | ***X*** | O | O |
| 7 | Was the methodology for data abstraction (including the value of health states and other benefits) stated? | O | O | O | O | O | O | O | O | O | O | O | O | O | O | O | O | O |
| 8 | Did the analytic horizon allow time for all relevant and important outcomes? Were benefits and costs that went beyond 1 year discounted (3% to 5%) and the justification given for the discount rate? | O | O | O | O | O | O | O | O | O | O | O | △ | O | O | X | O | O |
| 9 | Was the measurement of costs appropriate and the methodology for estimating quantities and unit costs clearly described? | O | O | O | O | O | O | O | O | O | O | O | O | O | O | O | O | O |
| 10 | Were the primary outcome measure(s) for the economic evaluation clearly stated, and did they include the major short-term, was justification given for the measures/scales used? | O | O | O | O | O | O | O | O | O | O | O | O | O | O | O | O | O |
| 11 | Were the health outcomes measures/scales valid and reliable? If previously tested valid and reliable measures were not available, was justification given for the measures/scales used? | O | O | O | O | O | O | O | O | O | O | O | O | O | O | O | O | O |
| 12 | Were the economic model (including structure), study methods and analysis, and the components of the numerator and denominator displayed in a clear, transparent manner? | O | O | O | O | O | O | O | X | O | O | O | O | O | O | O | O | O |
| 13 | Were the choice of economic model, main assumptions, and limitations of the study stated and justified? | O | O | O | O | O | O | O | X | O | O | O | O | O | O | O | O | O |
| 14 | Did the author(s) explicitly discuss the direction and magnitude of potential biases? | O | O | O | O | O | O | O | O | O | O | O | O | O | O | O | O | O |
| 15 | Were the conclusions/recommendations of the study justified and based on the study results? | O | O | O | O | O | O | O | O | O | O | O | O | O | O | O | O | O |
| 16 | Was there a statement disclosing the source of funding for the study? | O | O | O | O | ***X*** | O | O | O | O | O | O | O | O | O | O | O | O |
|  | Total Score | 100 | 100 | 100 | 100 | 97 | 100 | 100 | 85 | 100 | 100 | 100 | 96.5 | 100 | 100 | 85 | 100 | 100 |

QHES, Quality of Health Economic Studies; NA, Not available; O, Yes; △, Partial; *X*, No.
